# A Preliminary Study on the Development of a Brief Digital Literacy Scale for Older Adults

**DOI:** 10.1002/alz70857_104818

**Published:** 2025-12-25

**Authors:** Eun Hyun Seo, Hyung‐Jun Yoon, Seung Gon Kim, Ji Yeon Chung, Hoowon Kim, Kun Ho Lee

**Affiliations:** ^1^ Premedical Science, College of Medicine, Chosun University, Gwangju, Korea, Republic of (South); ^2^ Gwangju Alzheimer's & Related Dementias (GARD) Cohort Research Center, Gwangju, Korea, Republic of (South); ^3^ College of Medicine, Chosun University, Gwangju, Korea, Republic of (South); ^4^ Department of Neuropsychiatry, Chosun University Hospital, Gwangju, Korea, Republic of (South); ^5^ Department of Neurology, School of Medicine, Chosun University/Chosun University Hospital, Gwangju, Korea, Republic of (South); ^6^ Gwangju Alzheimer's Disease and Related Dementia Cohort Research Center, Chosun University, Gwangju 61452, Korea, Republic of (South)

## Abstract

**Background:**

The use of digital cognitive assessment tools has been rapidly increasing. However, digital literacy—a potential influential factor on performance with such tools—is often overlooked, and existing digital literacy scales are overly complex and time‐consuming. To address this gap, we developed a Brief Digital Literacy Scale (BDLS) specifically tailored for older adults to provide a simple pre‐assessment tool for digital cognitive evaluations.

**Method:**

The BDLS consists of 10 items measuring fundamental digital skills and frequency, scored on a 5‐point Likert scale. A total of 202 older adults participated in this study, including individuals with cognitive normal (CN), mild cognitive impairment (MCI), and Alzheimer's disease (AD) from the Gwangju Alzheimer's Disease and Related Dementia Cohort in Korea. Internal consistency and exploratory factor analysis (EFA), regression and partial correlation analysis were performed.

**Result:**

The BDLS demonstrated excellent internal consistency (α=0.874). EFA revealed a three‐factor structure, explaining 70.73% of the total variance: Factor 1: **Technical Proficiency**, Factor 2: **Smart Device Usage**, and Factor 3: **Digital Usage Frequency and Information Evaluation**. Higher BDLS scores were significantly associated with younger age, and longer years of education. Gender differences were not significant, though women showed a trend toward higher scores. BDLS scores showed significant partial correlations (controlling for age, gender, education) with: Boston Naming Test (*r* = 0.36, *p* <0.001), Visual Memory Test scores (*r* = 0.26∼0.37, *p* <0.01), Psychomotor Speed (*r* = −0.24, *p* <0.05). Significant associations were also observed with digital cognitive scores measuring visual memory (*r* = 0.19∼0.28, *p* <0.05∼ *p* <0.001), and processing speed (*r* = ‐0.23∼‐0.15, *p* <0.05 ∼ *p* <0.01). BDLS scores significantly differed across cognitive status groups (F=9.71, *p* <0.001), with scores decreasing progressively from CN to MCI to AD.

**Conclusion:**

Our preliminary results suggest that the BDLS is a reliable and valid tool for assessing digital literacy in older adults. It demonstrates relationships with cognitive performance (both paper‐and‐pencil and digital format). Notably, BDLS scores were significantly associated with visual memory and naming, highlighting the role of visual information processing and memory in digital literacy. This scale may provide a practical solution for pre‐assessment in digital cognitive testing and highlights the importance of considering digital literacy in clinical and research contexts.

